# Multi-spatial-attention U-Net: a novel framework for automated gallbladder segmentation on CT images

**DOI:** 10.1186/s12880-025-01737-7

**Published:** 2025-05-30

**Authors:** Henan Lou, Xiaobo Wen, Fanxia Lin, Zhan Peng, Qiuxiao Wang, Ruimei Ren, Junlin Xu, Jinfei Fan, Hao Song, Xiaomeng Ji, Huiyu Wang, Xiangyin Sun, Yinying Dong

**Affiliations:** 1 Department of Radiation Oncology, The Affiliated Hospital of Qingdao University, Qingdao, China; 2 Department of Radiology, The Affiliated Hospital of Qingdao University, Qingdao, China; 3 The Affiliated Hospital of Qingdao University, Qingdao University, Qingdao, China; 4 School of Pharmacy, Qingdao University, Qingdao, China; 5 Qingdao Cancer Institute, Qingdao University, Qingdao, China; 6 Department of Radiology, People’s Hospital of Rizhao, Rizhao, China; 7 Department of Radiation Oncology, Qingdao Chengyang District People’s Hospital, Qingdao, China; 8 Department of Oncology, Qingdao Sixth People’s Hospital, Qingdao, China

**Keywords:** Deep learning, Gallbladder, Automated delineation, U-Net, Multi-scale spatial attention

## Abstract

**Objective:**

This study aimed to construct a novel model, Multi-Spatial Attention U-Net (MSAU-Net) by incorporating our proposed Multi-Spatial Attention (MSA) block into the U-Net for the automated segmentation of the gallbladder on CT images.

**Methods:**

The gallbladder dataset consists of CT images of retrospectively-collected 152 liver cancer patients and corresponding ground truth delineated by experienced physicians. Our proposed MSAU-Net model was transformed into two versions V1(with one Multi-Scale Feature Extraction and Fusion (MSFEF) module in each MSA block) and V2 (with two parallel MSEFE modules in each MSA blcok). The performances of V1 and V2 were evaluated and compared with four other derivatives of U-Net or state-of-the-art models quantitatively using seven commonly-used metrics, and qualitatively by comparison against experienced physicians’ assessment.

**Results:**

MSAU-Net V1 and V2 models both outperformed the comparative models across most quantitative metrics with better segmentation accuracy and boundary delineation. The optimal number of MSA was three for V1 and two for V2. Qualitative evaluations confirmed that they produced results closer to physicians’ annotations. External validation revealed that MSAU-Net V2 exhibited better generalization capability.

**Conclusion:**

The MSAU-Net V1 and V2 both exhibited outstanding performance in gallbladder segmentation, demonstrating strong potential for clinical application. The MSA block enhances spatial information capture, improving the model’s ability to segment small and complex structures with greater precision. These advantages position the MSAU-Net V1 and V2 as valuable tools for broader clinical adoption.

**Supplementary Information:**

The online version contains supplementary material available at 10.1186/s12880-025-01737-7.

## Introduction

The gallbladder, a small pear-shaped organ located beneath the liver, plays a significant role in the digestive system by storing and concentrating bile, which is essential for fat emulsification and nutrient absorption [1–3]. Precise gallbladder segmentation is of extreme importance in liver cancer treatment, where the gallbladder often becomes involved [4, 5]. Accurate gallbladder delineation can assist surgeons in surgical planning for liver cancer surgery, avoiding damage to the gallbladder during the surgical process. Additionally, accurate delineating the gallbladder as organs at risk(OAR) in liver cancer treatment can effectively reduce radiation damage to the gallbladder and its surrounding structures, thereby decreasing or avoiding the probability of adverse reactions and further improving patients’ life quality.

In the clinical practice, segmentation is commonly and manually conducted by physicians, which is time-consuming and suffers from inter- and intra-observer variability. In recent years, deep learning-based methods, particularly U-Net and its variants, have emerged as powerful tools for medical image segmentation [6–8], which has promoted substantial effective application and extension of deep learning based automatic delineation in clinical settings. Oktay et al. [9]. proposed an Attention U-Net and applied it into pancreatic segmentation. The Attention U-Net demonstrated excellent segmentation performance with good efficiency. Girl et al. [10]. employed the U-Net for automatic liver segmentation. The results revealed that the model exhibited strong performance with capability of tackling the difficulties in precisely distinguishing the liver on abdominal CT scans while Seenia Francis et al. [11] proposed a deep learning model based on a generative adversarial network for auto-contouring of OARs on abdominal CT images and obtained significantly improved segmentation. The obtained results proved that the suggested model was able to compete with existing state-of-the-art abdominal OAR segmentation techniques. Liao et al. [12]. developed a deep learning model, namely AbsegNet, for the segmentation of 16 OARs in the abdomen. The results indicated that most of the contours generated by AbsegNet were accurate and robust, exhibiting good clinical applicability.

However, current deep learning approaches still confront such tremendous challenges as under-and-over segmentation of small or irregularly shaped organs like the gallbladder, sensitivity to variations in image quality, and a need for massive labeled data sets, which are often scarce, labor-intensive and access-limited. Despite gallbladder’s importance in surgical planning and radiation therapy, a large proportion of researches on deep learning-based segmentation of abdominal organs have ignored the gallbladder, often overlooking its critical role in ensuring the efficacy and safety of treatments [13–16]. The evident need for advanced computational models that can consistently and accurately segment the gallbladder is prompting further research and development in this area.

Our study introduces a new multi-spatial attention mechanism block, which can capture and integrate features accurately at multiple scales, and incorporates it into the U-Net to construct a novel deep learning framework, aiming to enhance gallbladder segmentation so as to improve surgical planning and the accuracy of radiotherapy for patients with liver cancer and related tumors, ultimately enhancing clinical outcomes, optimizing treatment duration, and reducing associated side effects. Specifically, our contributions are two:

a. Higher capability of capturing spatial features.

We proposed a multi-scale spatial attention (MSSA) module, which encourages the model to acquire spatial information at different scales and dynamically weighting the gallbladder region at various spatial resolutions while suppressing irrelevant backgrounds, promoting the accuracy of the model.

b. Better information fusion.

We embedded a multi-scale feature extraction and fusion (MSFEF) module, which employs convolutions with different kernel sizes to effectively capture both global and local information of the gallbladder.

## Materials and methods

### Data collection

**Fig. 1:**
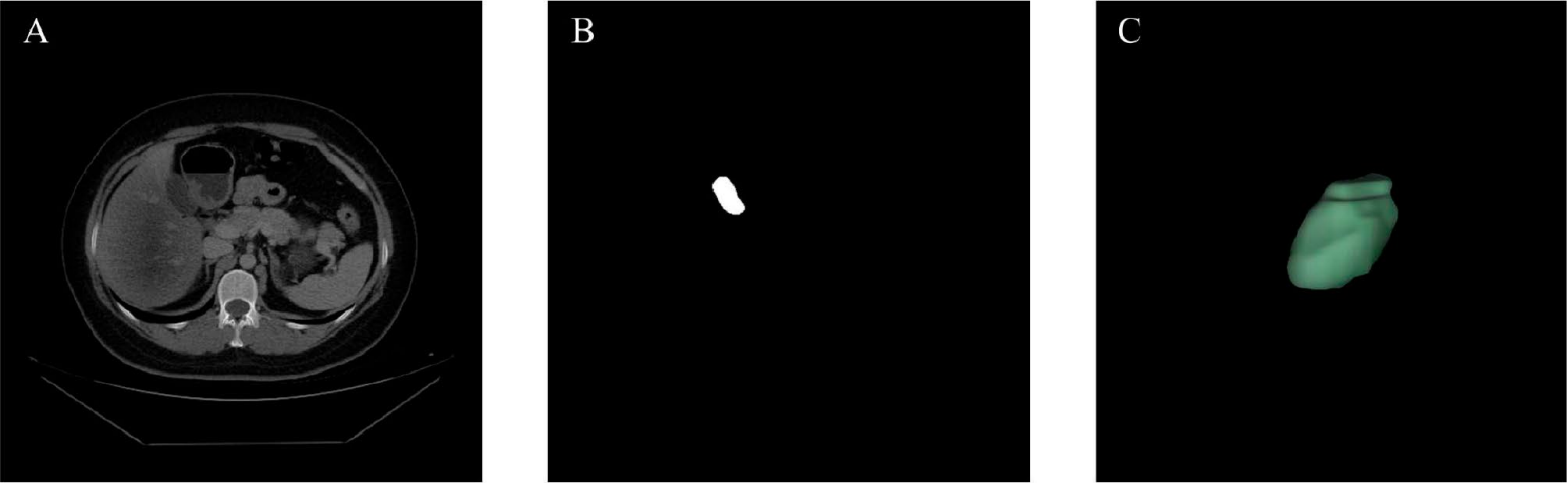
Dataset (**A**). CT image (**B**). Ground truth. (**C**). Gallbladder by 3D

The dataset consists of CT images of 152 liver cancer patients, retrospectively collected from Qingdao University Affiliated Hospital, and corresponding ground truth delineated by experienced physicians (As shown in Fig. 1). The CT examination was conducted using a multidetector CT scanner comprising Optima CT670 from GE Healthcare, iCT 256 from Philips Healthcare, and SOMATOM Definition Flash from Siemens Medical Systems. Inclusion criteria: **a)**. The CT image must contain a complete gallbladder. **b).** The gallbladder should be clearly visible. **c).** Scans were performed following standard clinical protocols for liver cancer staging. Our study utilized enhanced imaging to ensure optimal visualization of the liver and its surrounding anatomical structures, including the gallbladder. Specific CT scan parameters and enhancement methods are provided in the supplementary materials Part 1.

The dataset was divided into three sets: a training set (70 cases), a validation set (11 cases), and a test set (71 cases) following an approximate ratio of 7:1:7. This partitioning was designed to ensure the model’s robustness, avoid overfitting and preserve adequate data for model evaluation. Data augmentation was performed to the training set in response to inadequate data availability and the high costs associated with obtaining accurate mask images. Specifically, rotation, shift, shear, and zoom were employed to enhance the model’s adaptability to variations in orientation, positional shifts (both horizontal and vertical), non-uniform deformations, and scale differences. These transformations were systematically applied to simulate the inherent variations that occur during medical image acquisition and positioning, whereby the model gained significantly improved generalization. Detailed augmentation parameters are provided in the supplementary materials Part 2.

### Data preprocessing

To prepare the CT images for model training, a series of preprocessing steps were performed. First, Hounsfield Unit (HU) conversion was applied to standardize pixel intensities, transforming them into HU values. Window width and level adjustments were then applied to enhance the visibility of the liver and gallbladder regions, with windowing parameters set specifically for liver and gallbladder tissue contrast optimization. Next, adaptive histogram equalization was employed to improve the contrast within the region of interest (ROI), further enhancing the visibility of key anatomical structures, especially in the case of low contrast. These preprocessing techniques allowed for a more consistent input to the segmentation model, improving model performance by enhancing the clarity of ROI in each scan.

### Model framework

**Fig. 2:**
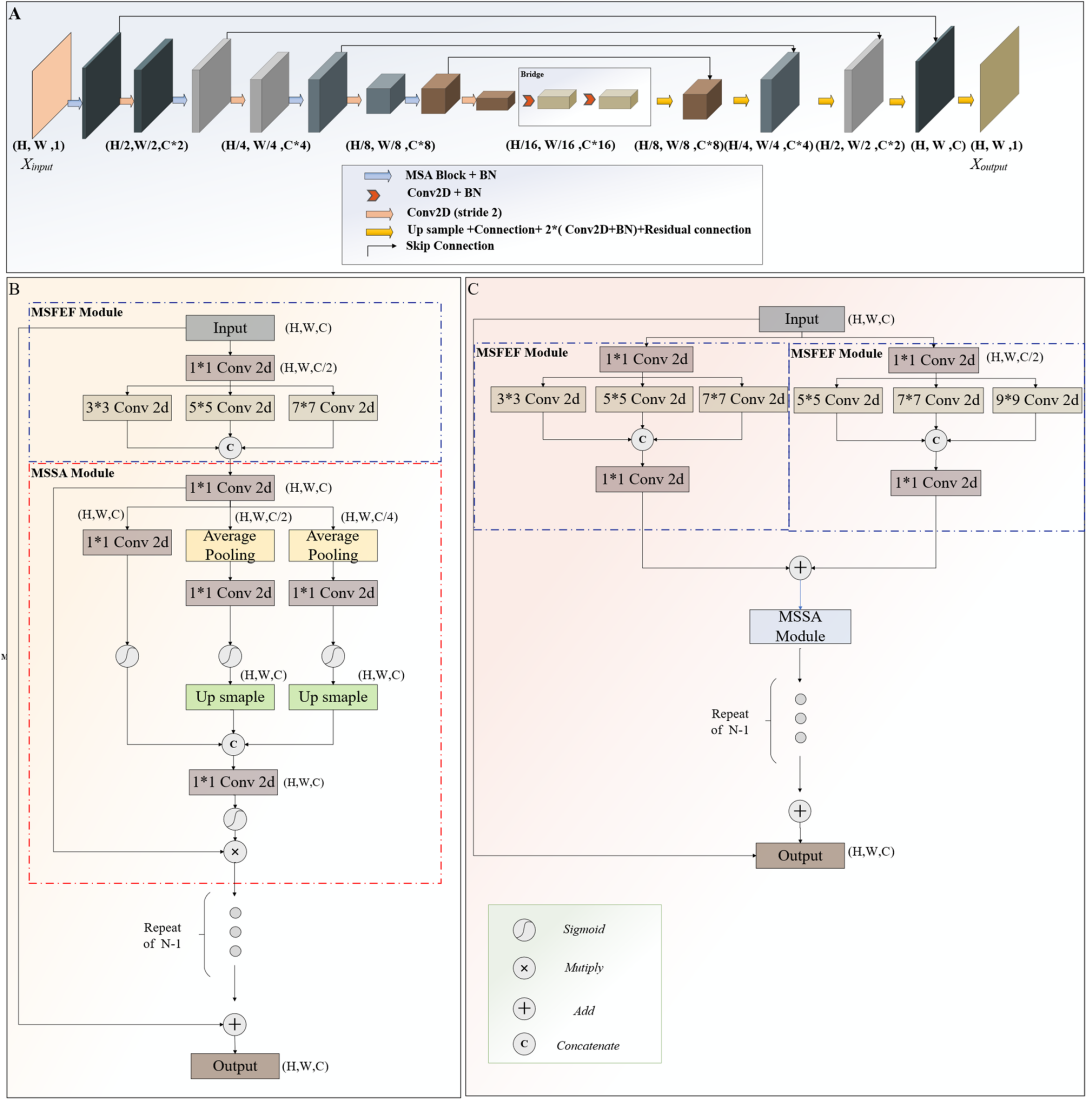
Frameworks **(A)** MSAU-Net Framework **(B)** MSA block in MSAU-Net V1 **(C)** MSA block in MSAU-Net V2

The U-Net model [17] is extensively applied in automatic medical image segmentation due to its distinctive architecture, encoder-decoder structure and skip connections, which ensures its superior segmentation in the case of limited training data. However, its single convolutional kernel size restricts its ability to capture multi-scale features, thus provoking the failure to effectively combine global and local information, especially in the tasks with complex and fine structures, and impairing the model’s segmentation accuracy. In response to these limitations, we proposed a novel deep learning block-Multi-Spatial-Attention (MSA) Block, and embedded it into the U-net architecture, obtaining the **MSAU-Net**, designed to improve the model’s competence of capturing multi-scale features and fine structures through multi-scale feature extraction and attention mechanisms in pursuit of improved gallbladder segmentation, as shown in Part A of Fig. 2. Source code has been provided at https://github.com/XiaoboWen-AI/Multi-Spatial-Attention-U-Net. The premier innovations of the MSAU-Net is the incorporation of Multi-Spatial Attention (MSA) block, which includes Multi-Scale Feature Extraction and Fusion Module (MSFEF) and Multi-Scale Spatial Attention Module (MSSA). The detailed description is as follows:

#### Multi-scale feature extraction and fusion module

MSFEF utilizes multi-scale convolutional kernels (3, 5, 7 or 5, 7, 9) to extract features of different scales, and these extracted features are subsequently fused into feature maps with multi-scale information. Such MSFEF offers two advantages: (*i*) Convolutional kernels of different scales enables the models to acquire multi-scale feature information, thereby obtaining both global contextual information and local details more effectively, which is crucial for accurately segmenting anatomical structures of varying scales. (*ii*) The integration of global and local information strengthens the model’ ability to maintain global consistency as well as capture complex details and contextual relationships, thereby improving the model’s segmentation performance.

#### Multi-Scale Spatial attention module

The similarity in gray-scale values between the gallbladder and adjacent organs, as well as its small size and irregular shape, provoke difficulty for boundary distinguishment. Therefore, it is critical to capture spatial context and detailed structural features around the gallbladder for improving segmentation accuracy.

For this issue, channel squeeze and spatial Excitation (sSE) mechanism is commonly used in the present study, so initially, we employed sSE mechanism to enhance spatial feature recalibration [18]. MSAU-Net with sSE (as shown in the supplementary materials Part 3) exhibited some improvements than U-Net, but its segmentation is still unsatisfactory because the single-scale nature of sSE limited the model’s ability to fully capture complex and essential multi-scale spatial features, consequently affecting segmentation accuracy.

To overcome the constraints of sSE, we propose a novel attention mechanism—Multi-Scale Spatial Attention (MSSA), as illustrated in Part B of Fig. 2. The MSSA mechanism utilizes two average pooling layers to downsample the input features, generating feature maps at different scales, which facilitates the model’s capability of capturing multi-scale spatial features. Subsequently, a Conv2D layer and a Sigmoid activation function are applied to calculate the spatial attention weights for each scale. These attention weights represent the relative importance of spatial features at different resolutions. The downsampling attention maps are then upsampled back to the original input size and fused with the features obtained from the sSE mechanism, producing an initial set of multi-scale spatial attention weights. Next, another Conv2D layer and Sigmoid activation function are applied to generate the final multi-scale spatial attention weights, which are multiplied element-wise with the original input feature map to recalibrate the spatial features, effectively enhancing the model’s ability to focus on the most relevant regions at different scales.

Compared to the single-scale sSE attention mechanism, MSSA attention module innovatively utilizes multi-scale feature extraction to capture spatial dependencies between different scales, which enables the model to more effectively handle the complex spatial structures such as gallbladder and its surrounding tissues. Furthermore, the integration of multi-scales spatial attention leverages both single-scale and multi-scale spatial information to provide a more comprehensive attention mechanism.

#### Other improvements and variants of the MSAU-Net

We have inserted a residual shortcut connection between the input and the final output of the MSA Block to avoid gradient explosion and vanishing and further enhance feature propagation [19]. Additionally, the introduction of residual connections further stabilizes the training process and allows for deeper model architectures, enabling the model to incorporate more multi-scale feature extraction modules. This facilitates the extraction of a richer set of features, thereby improving the model’s ability to capture complex anatomical structures.

Additionally, we have optimized our proposed model by improving the upsampling section of the traditional U-Net model. We connected a Batch Normalization (BN) layer [20] after each convolutional layer for the stabilization of the training process, prevention from gradient vanishing or explosion, and accelerated convergence speed. Furthermore, residual connections were incorporated, which further enhanced the information flow capability while further resolving the issue of gradient vanishing or explosion.

Based on the configuration of the MSA Block, this study proposed two versions of the MSAU-Net, referred to as MSAU-Net V1 and MSAU-Net V2 respectively. As illustrated in Part B of Fig. 2, in V1, the model employs only one MSFEF module (with kernels size being 3,5,7), which is connected with an MSSA module. Different from V1, as shown in Part C of Fig. 2, V2 adopts two parallel MSFEF modules (with one convolution kernels size being 3, 5, 7 and the other 5, 7, 9), also connected with an MSSA module. Detailed hyperparameters and environmental configurations of the models are provided in supplementary materials Part 4&Part 5.

### Loss function and evaluation metrics

In traditional deep learning-based segmentation tasks, the cross-entropy loss function is commonly employed due to its pixel-wise classification ability [21–23]. However, cross-entropy treats all pixels equally, which makes the model tend to focus more on the background, leading to unsatisfactory segmentation in tasks with small target regions, such as gallbladder. To address this issue, we employed a more suitable loss function for small medical datasets of small targets-**Dice loss (DL) function**, which is designed to handle class imbalance and better emphasize small target regions [24]. 

DL loss function facilitates the model to focus more effectively on the critical structures, provoking improved segmentation accuracy, especially in the cases where the target organ occupies only a small portion of the image. The formula is as follows

1 $$Dic{e_{loss}} = 1 - 2*\frac{{\left| {GT \cap \Pr ed} \right| + \varepsilon }}{{\left| {GT} \right| + \left| {\Pr ed} \right| + \varepsilon }}$$

where GT represents the ground truth, and Pred denotes the predicted segmentation produced by the model. The term $\varepsilon$ is a small constant, included to avoid division by zero.

To comprehensively and quantitatively evaluate the performance of the model, this study employed a series of evaluation metrics, categorized into two main groups. The first group assesses classification accuracy- the overlap between predicted regions and ground truth-including the Dice Similarity Coefficient (DSC), Jaccard Similarity Coefficient (JSC), Positive Predictive Value (PPV), and Sensitivity (SE). The second group encompasses Hausdorff Distance (HD), Relative Volume Difference (RVD), and Volumetric Overlap Error (VOE), which focuses on evaluating boundary accuracy and volumetric differences. Detailed formulas can be found in the supplementary materials Part 6.

### Comparison model design

Comparative experiments were performed to prove the validity of the proposed MSAU-Net model with U-Net and Attention U-Net, TransUNet, Swin-Unet as the control group.

- **U-Net** [17]: U-Net, the most widely-used model in medical image segmentation, consists of upsampling, downsampling, and skip connection. Downsampling extract feature information of the target while the upsampling gradually restore spatial resolutions. The skip connection conveys the features obtained during downsampling to the corresponding layers during upsampling, which compensates for the loss of high-resolution features in the downsampling process, leading to improved segmentation accuracy.
- **Attention U-Net** [9]: Attention U-Net incorporates an attention mechanism into the skip connections of the traditional U-Net. The attention gate weights the feature maps conveyed by the downsampling, thereby capturing critical features of the target while suppressing irrelevant background information. This adaptive feature selection enhances the segmentation accuracy of the model when dealing with complex structures.
- **TransUNet** [25]: Traditional convolutional operations are limited in long-range dependency modeling. To handle the issue, TransUNet adopts a hybrid CNN-Transformer architecture with incorporation of Transformer modules into the U-Net architecture. After upsampling the encoded features, the decoder combines them with high-resolution CNN feature maps for precise localization, thereby boosting the model’s segmentation accuracy for the target.
- **Swin-Unet** [26]: Swin-Unet, which is a U-Net-like pure Transformer for medical image segmentation.
  It replaces traditional convolutional operations with a hierarchical Swin Transformer with shifted windows as the encoder to extract contextual features, balancing global context modeling and local detail preservation. And a symmetric Swin Transformer-based decoder with patch expanding layer is designed to perform the up-sampling operation to restore the spatial resolution of the feature maps, which allows for better coordination of global information with local details, achieving precise segmentation.

### Statistical analysis

In this study, descriptive statistics for quantitative results were presented using means and standard deviations. One-way analysis of variance (ANOVA) was conducted among groups to assess whether there were significant differences among them. If the ANOVA results indicated significant group differences, the least significant difference (LSD) was further utilized for pairwise comparisons to detect which specific group differences are statistically significant. A P-value of smaller than 0.05 was considered indicative of a statistically significant difference.

## Results

### Quantitative analysis of the modules

We conducted related experiments to determine the optimal numbers of MSA block for different versions of MSAU-Net, namely MSAU-Net V1 with sSE, MSAU-Net V1 with MSSA, MSAU-Net V2 with sSE and MSAU-Net V2 with MSSA. Detailed information is provided in Supplementary materials Part 7.

**Fig. 3:**
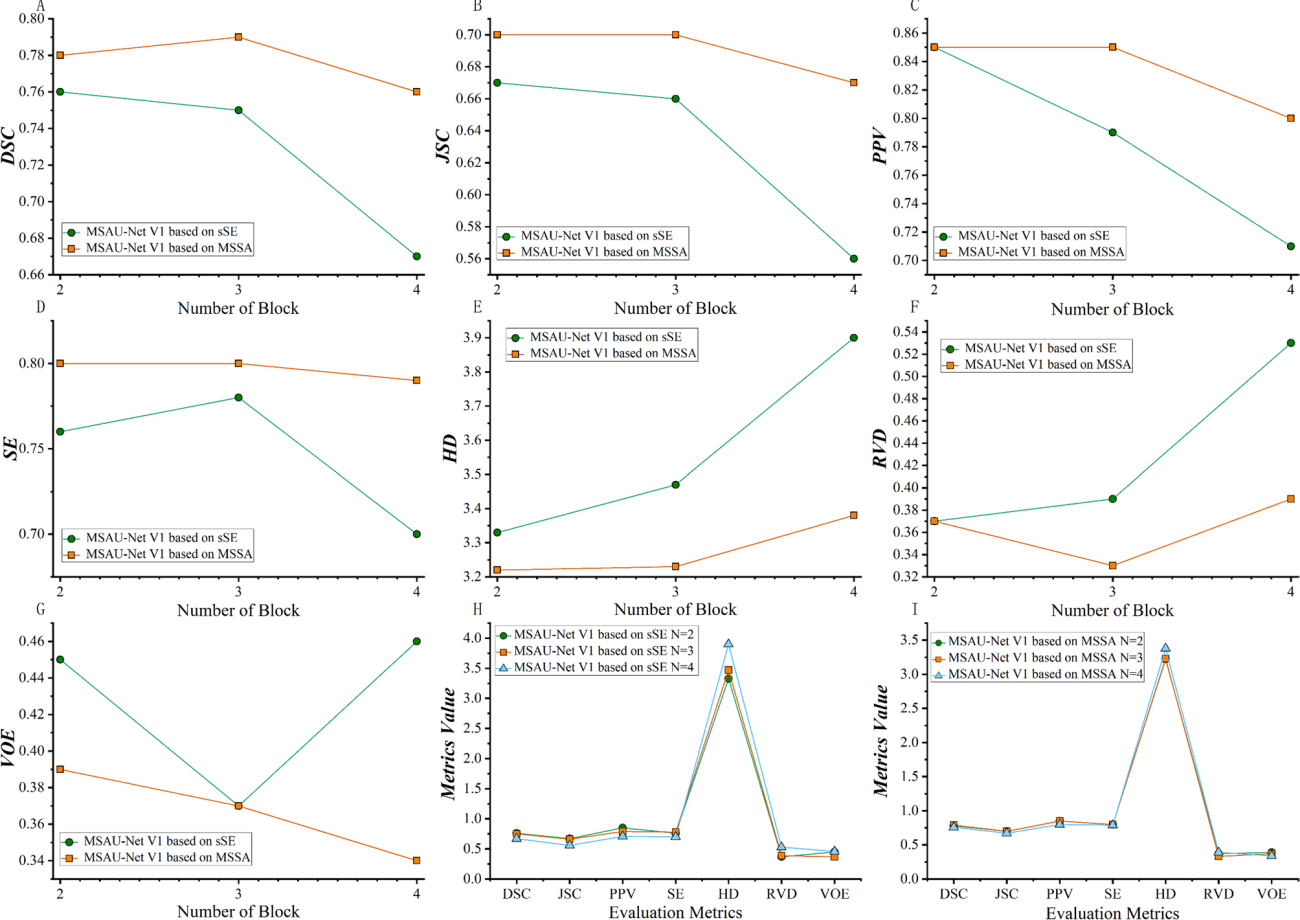
Quantitative analysis of MSAU-Net V1with different numbers of MSA block. From A to G, green line stands for V1 with sSE and orange line stands for V1 with MSSA. **(A)** DSC. **(B)** JSC. **(C)** PPV **(D)** SE **(E)** HD. **(F)**. RVD **(G)**. VOE **(H)** Quantitative evaluation index of sSE attention mechanism with different numbers of MSA block. **(I)** Quantitative evaluation index of MSSA attention mechanism with different numbers of MSA block)

**Fig. 4:**
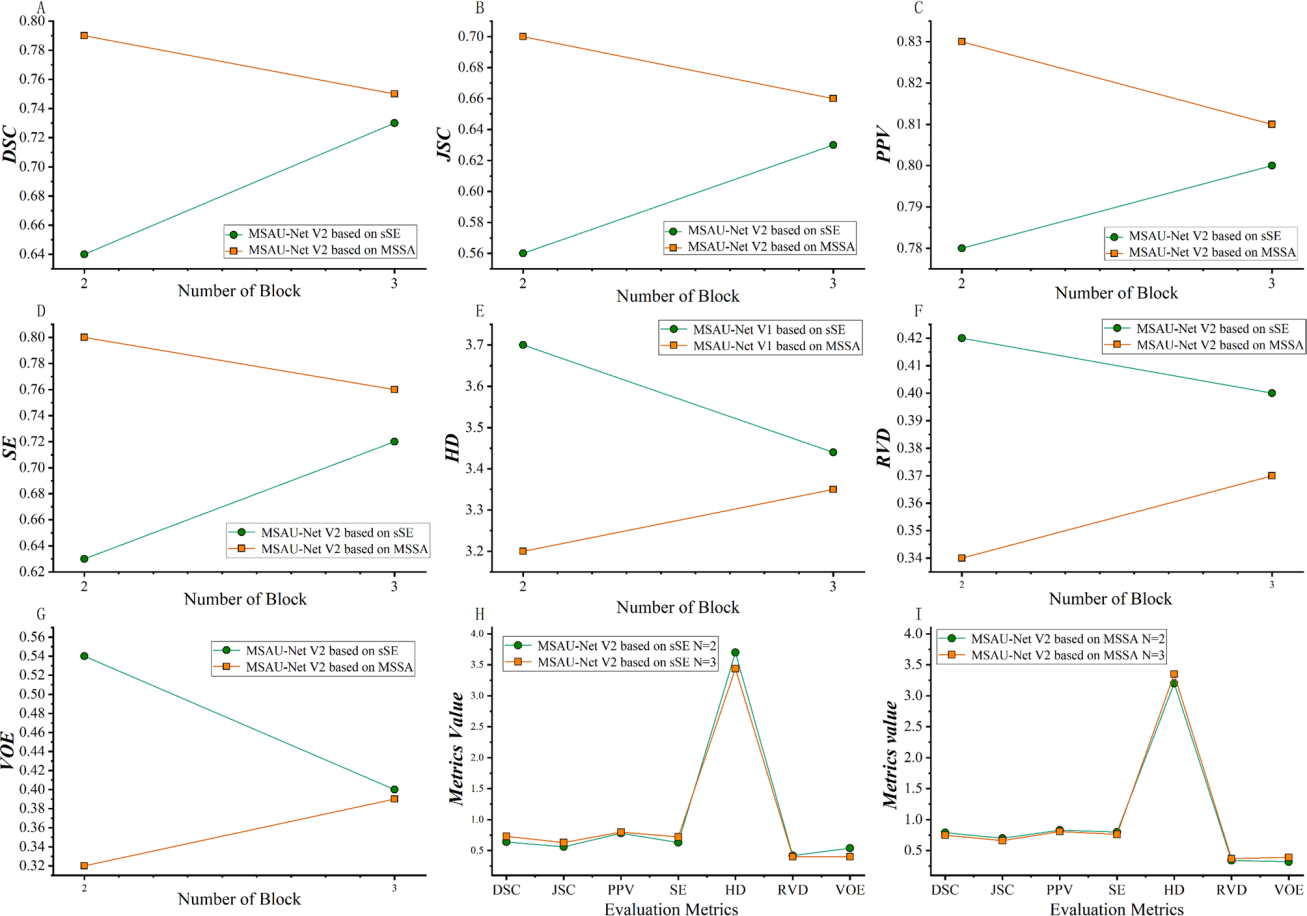
Quantitative analysis of MSAU-Net V2 with different numbers of MSA block. From A to G, green line stands for V2 with sSE and orange line stands for V2 with MSSA. **(A).** DSC. **(B).** JSC. **(C).** PPV **(D).** SE **(E).** HD. **(F)**. RVD **(G)**. VOE **(H).** Quantitative evaluation index of sSE attention mechanism with different numbers of MSA block. **(I).** Quantitative evaluation index of MSSA attention mechanism with different numbers of MSA block)

For the MSAU-Net V1(sSE mechanism), optimal performance was achieved with two MSA blocks, which exhibited superior properties in several metrics. For MSAU-Net V1 (MSSA mechanism), three MSA blocks presented best performance, as shown in Fig. 3. For MSAU-Net V2 (sSE), the architecture with three MSA blocks showed the best results while for MSAU-Net V2 (MSSA), a two-module architecture was optimal, as shown in Fig. 4.)

Subsequently, we conducted comparative experiments to validate the effectiveness of MSSA. We employed the four models to perform segmentation on the training set. As shown in Table 1, the results revealed that, compared with MSAU-Net V1 with sSE, MSAU-Net V1 with MSSA increased DSC, JSC and SE by 0.03, 0.03and 0.04 respectively and decreased HD, RVD and VOE by 0.10, 0.06 and 0.09 respectively. Similarly, compared with MSAU-Net V2 (sSE), MSAU-Net V2 (MSSA) increased DSC, JSC and SE by 0.06, 0.07 and 0.08 respectively and decreased HD, RVD and VOE by 0.24, 0.07 and 0.08 respectively.

**Table 1:** Quantitative comparison of the four models for MSSA validation ($\overline x \pm s$)

	DSC	JSC	PPV	SE	HD	RVD	VOE
MSAU-Net V1 (sSE)	0.76 ± 0.27	0.67 ± 0.28	0.85 ± 0.20	0.76 ± 0.29	3.33 ± 1.29	0.32 ± 0.49	0.38 ± 0.59
MSAU-Net V1 (MSSA)	**0.79 ± 0.25**	**0.70 ± 0.25**	**0.85 ± 0.20**	**0.80 ± 0.26**	3.23 ± 1.23	**0.26 ± 0.40**	0.29 ± 0.47
MSAU-Net V2 (sSE)	0.73 ± 0.28	0.63 ± 0.28	0.80 ± 0.26	0.72 ± 0.28	3.44 ± 1.23	0.34 ± 0.47	0.35 ± 0.46
MSAU-Net V2 (MSSA)	**0.79 ± 0.24**	**0.70 ± 0.25**	0.83 ± 0.23	**0.80 ± 0.25**	**3.20 ± 1.23**	0.27 ± 0.45	**0.27 ± 0.40**

Note: The values in bold represent the highest scores achieved

In sum, the replacement of sSE with MSSA enhances the model’s capability of capturing and utilizing multi-scale spatial information, significantly improving the model’s ability to segment the gallbladder on CT images, and exhibiting superiority over the single-scale spatial attention mechanism.

In view of superiority of MSSA over sSE, we adopted MSSA module in MSA block in the following model comparison with the control group.

### Quantitative analysis of the model comparison

As detailed in Table 2, the quantitative metrics revealed that the proposed MSAU-Net V1 and V2 outperformed the four comparative models.

**Table 2:** Quantitative comparison of our proposed models and control group ($\overline x \pm s$)

	DSC 95%CI	JSC 95%CI	PPV 95%CI	SE 95%CI	HD 95%CI	RVD 95%CI	VOE 95%CI
U-Net	0.72 ± 0.33^*#^ [0.69,0.75]	0.64 ± 0.32^*#^ [0.62,0.67]	0.78 ± 0.34^*#^ [0.75,0.82]	0.70 ± 0.33^*#^ [0.67,0.73]	3.36 ± 1.45 [3.22,3.49]	0.33 ± 0.50^*#^ [0.29,0.38]	0.43 ± 0.62^*#^ [0.38,0.49]
Attention U-Net	0.75 ± 0.30^*^ *[0.73*,*0.78]*	0.67 ± 0.30 [0.65,0.70]	0.86 ± 0.20 [0.84,0.87]	0.76 ± 0.31^*^ [0.73,0.79]	3.29 ± 1.36 [3.16,3.41]	0.30 ± 0.44 [0.26,0.34]	0.38 ± 0.59^*#^ [0.33,0.44]
TransUNet	0.69 ± 0.26^*#^ [0.67,0.72]	0.58 ± 0.26^*#^ [0.56,0.61]	0.69 ± 0.26^*#^ [0.67,0.71]	0.79 ± 0.26 [0.77,0.81]	3.99 ± 1.37^*#^ [3.87,4.11]	0.59 ± 0.89^*#^ [0.51,0.67]	0.42 ± 0.44^*#^ [0.38,0.46]
Swin-Unet	0.62 ± 0.30^*#^ [0.59,0.65]	0.51 ± 0.28^*#^ [0.49,0.54]	0.73 ± 0.30^*#^ [0.71,0.76]	0.61 ± 0.32^*#^ [0.58,0.64]	3.93 ± 1.23^*#^ [3.82,4.04]	0.44 ± 0.51^*#^ [0.39,0.48]	0.50 ± 0.54^*#^ [0.45,0.55]
MSAU-Net V1 (MSSA)	0.79 ± 0.25 [0.77,0.81]	0.70 ± 0.25 [0.68,0.73]	0.85 ± 0.20 [0.83,0.87]	0.80 ± 0.26 [0.78,0.82]	3.23 ± 1.23 [3.12,3.35]	0.26 ± 0.40 [0.23,0.30]	0.29 ± 0.47 [0.25,0.34]
MSAU-Net V2 (MSSA)	0.79 ± 0.24 [0.77,0.81]	0.70 ± 0.25 [0.68,0.72]	0.83 ± 0.23 [0.81,0.86]	0.80 ± 0.25 [0.77,0.82]	3.20 ± 1.23 [3.09,3.31]	0.27 ± 0.45 [0.23,0.31]	0.27 ± 0.40 [0.24,0.31]
***F***	25.34	35.14	21.87	31.00	36.22	25.49	13.33
***P***	***< 0.001***	***< 0.001***	***< 0.001***	***< 0.001***	***< 0.001***	***< 0.001***	***< 0.001***

Note: F represents Fisher’s F-statistic in ANOVA (ratio of between-group to within-group variation), and p denotes the significance probability. * denotes the significant difference between MSAU-Net V1 and the model with *. # denotes the significant difference between MSAU-Net V2 and the model with #

Compared with the four comparative models, MSAU-Net V1 exhibited increased competence for accurate segmentation of complex anatomical structures. It obtained 6 optimal metrics: DSC of 0.79 ± 0.25, JSC of 0.70 ± 0.25, SE of 0.80 ± 0.26, HD of 3.23 ± 1.23 RVD of 0.26 ± 0.40 and VOE of 0.29 ± 0.47. Its PPV (0.85 ± 0.20), is second to that of Attention U-Net (0.86 ± 0.20). As shown in Supplementary materials Part 8, LSD analysis reveal that MSAU-Net V1 presents statistically significant difference from the four comparative models for DSC (U-Net: *p* < 0.001; Attention U-Net: *p* = 0.04; TransUNet: *p* < 0.001; Swin-Unet: *p* < 0.001) and VOE (U-Net: *p* < 0.001; Attention U-Net: *p* = 0.01; TransUNet: *p* < 0.001; Swin-Unet: *p* < 0.001). For JSC, PPV and RVD, MSAU-Net V1 presents significant difference from U-Net (p_JSC_< 0.001, p_PPV_ = 0.03 and p_RVD_ = 0.05), TranUNet (all three metrics *p* < 0.001) and Swin-Unet (all three metrics *p* < 0.001), but no significant difference from Attention U-Net (p_JSC_=0.09, p_ppv_ =0.14 and p_RVD_=0.32). For SE, MSAU-Net V1 shows significant difference from U-Net (*p* < 0.001), Attention U-Net (*p* = 0.03) and Swin-Unet (*p* < 0.001), but no significant difference from TransUNet (p_SE_ =0.58). For HD, MSAU-Net V1 shows significant difference from TransUNet (*p* < 0.001) and Swin-Unet (*p* < 0.001), but no significant difference from U-Net (p_HD_= 0.16) and Attention U-Net (p_HD_=0.55). Compared with the four comparative models, MSAU-Net V2 obtained 6 optimal metrics: DSC of 0.79 ± 0.24, JSC of 0.70 ± 0.25, SE of 0.80 ± 0.25, HD of 3.20 ± 1.23, RVD of 0.27 ± 0.45 and VOE of 0.27 ± 0.40. Its PPV (0.83 ± 0.23), is second to that of Attention U-Net (0.86 ± 0.20). As shown in Supplementary materials Part 8, LSD analysis reveal that MSAU-Net V2 presents statistically significant difference from all the comparative models for VOE (all *p* < 0.001). For DSC(all *p*< 0.001), JSC(all *p*< 0.001) and PPV(p_U−Net_=0.03, p_TransUnet_<0.001, p_Swin−Unet_<0.001), MSAU-Net V2 presents significant difference from the three other comparative models(except Attention U-Net: p_DSC_=0.06, p_JSC_=0.14 and p_PPV_=0.15). For HD and RVD, it shows significant difference from TransUNet (both *p* < 0.001) and Swin-Unet (both *p* < 0.001), but no significant difference from U-Net (p_HD_=0.06 and p_RVD_=0.10) and Attention U-Net (p_HD_=0.30 and p_RVD_=0.51). For SE, MSAU-Net V2 shows significant difference from U-Net (*p* < 0.001) and Swin-Unet (*p* < 0.001), but no significant difference from Attention U-Net (p_SE_=0.06) and TransUNet (p_SE_=0.81).

In summary, the MSAU-Net V1 and V2 both demonstrated significant improvements across most metrics compared to the comparison models. These results underscore the effectiveness of the MSA block in capturing the spatial structure and adjacent positioning of the gallbladder, thus significantly enhancing the segmentation accuracy.

### Qualitative analysis of the models’ performances

**Fig. 5:**
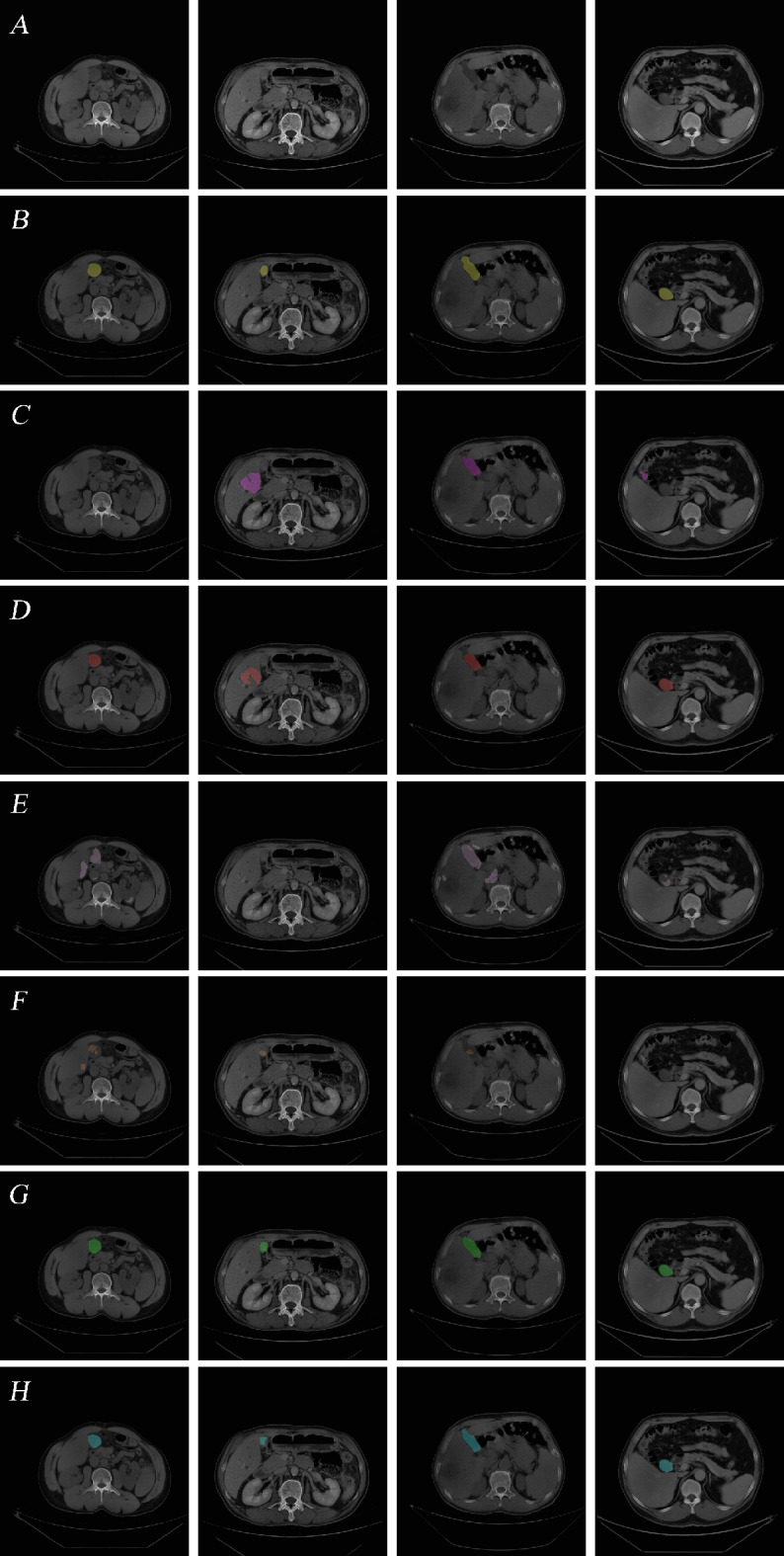
CT image and gallbladder segmentation by the six models (**A**) Original CT image. (**B**) Expert-delineated GT. (**C**) Segmentation by U-Net. (**D**) Segmentation by Attention U-Net. (**E**) Segmentation by TransUNet. (**F**) Segmentation by Swin-Unet (**G**) Segmentation by MSAU-Net V1. (**H**) Segmentation by MSAU-Net V2

The qualitative results are illustrated in Fig. 5, in which C1, C2, and C4 reveal the incompetence of U-Net model for precise segmentation of smaller structures and areas with low contrast. From C1, C2 and C4, we can detect wrongly-segmented parts and specific regions which are not segmented. The Attention U-Net shows some improvement than U-Net in handling these challenges, but it still exhibits problems such as scattered points and incorrect segmentations, as shown by D1, D4, and D2 in Fig. 5. What’s more, both the U-Net and Attention U-Net models display over-segmentation and under-segmentation in various instances, indicating a need for more stable and robust models for accurate gallbladder segmentation. Transformer-based TransUNet and Swin-Unet exhibit relatively unsatisfactory segmentation with distinct scatter points as well as over-segmentation and under-segmentation (as shown in 5E and 5 F). This may be attributable to insufficient training data because Transformer architectures typically demand substantial training data.

In contrast to the control group, the MSAU-Net V1 and V2 demonstrate substantial enhancements in addressing the segmentation challenges mentioned above. They effectively alleviate erroneous segmentation and segment the structures that the other models fail to, and, with clarity, they reduce both over-segmentation and under-segmentation. This advancement is attributed to the efficacy of MSA block in capturing and integrating multi-scale spatial information, which is critical in accurately delineating complex anatomical structures in medical images.

### External validation experiment

To further assess our proposed model’s generalization capability, we conducted an external validation experiment. The data used for the external validation experiments included CT images of 25 patients at *People’s Hospital of Rizhao* (CT scan parameters and enhancement methods can be seen in supplementary materials Part I) and these data were not used in the model training process, which ensures the objectivity of the experiment. The results are shown in Table 3.

**Table 3:** Quantitative comparison of our proposed models and comparison models($\overline x \pm s$)for external validation

	DSC	JSC	PPV	SE	HD	RVD	VOE
U-Net	0.61 ± 0.35 ^*#^	0.52 ± 0.33^*#^	0.74 ± 0.38^*#^	0.56 ± 0.35^*#^	4.29 ± 1.76	0.43 ± 0.35^***#***^	0.69 ± 0.71^*#^
Attention U-Net	0.67 ± 0.33	0.57 ± 0.31	0.82 ± 0.28^***#***^	0.64 ± 0.34	4.17 ± 1.73	0.37 ± 0.38	0.54 ± 0.65
TransUNet	0.65 ± 0.27^***#***^	0.53 ± 0.26^***#***^	0.73 ± 0.26^*#^	0.67 ± 0.31	4.59 ± 1.38^*#^	0.51 ± 0.72^*#^	0.53 ± 0.52
Swin-Unet	0.62 ± 0.29^***#***^	0.50 ± 0.27^*#^	0.78 ± 0.26^*#^	0.58 ± 0.30^***#***^	4.31 ± 1.46	0.39 ± 0.32	0.54 ± 0.57
MSAU-Net V1	0.67 ± 0.31	0.58 ± 0.30	0.89 ± 0.18	0.64 ± 0.33	4.09 ± 1.58	0.37 ± 0.33	0.55 ± 0.64
MSAU-Net V2	0.71 ± 0.28	0.61 ± 0.27	0.85 ± 0.22	0.68 ± 0.30	4.04 ± 1.49	0.32 ± 0.31	0.45 ± 0.56
***F***	2.99	4.02	4.21	4.32	3.07	4.43	3.00
***P***	***0.01***	***< 0.001***	***< 0.001***	***< 0.001***	***0.01***	***< 0.001***	***0.01***

Note: F represents Fisher’s F-statistic in ANOVA (ratio of between-group to within-group variation), and p denotes the significance probability. * denotes the significant difference between MSAU-Net V1 and the model with *. # denotes the significant difference between MSAU-Net V2 and the model with #

The experimental results showed that compared with control group, MSAU-Net V1 obtained 5 optimal metrics: DSC of 0.67 ± 0.31,JSC of 0.58 ± 0.30, PPV of 0.89 ± 0.18, HD of 4.09 ± 1.58 and RVD of 0.37 ± 0.33. TransUNet achieved the best SE of 0.67 ± 0.31 (MSAU-Net V1 obtained 0.64 ± 0.33)and the best VOE of 0.53 ± 0.52 (MSAU-Net V1 obtained 0.55 ± 0.64). As shown in Supplementary materials Part 8, LSD analysis shows that for DSC, SE and VOE, MSAU-Net V1 presents significant difference from U-Net(p_DSC_ = 0.04, p_SE_ = 0.02, and p_VOE_ = 0.03) but no significant difference from Attention U-Net(p_DSC_=0.78, p_SE_=0.92 and p_VOE_=0.89), TransUNet (p_DSC_=0.36, p_SE_=0.25 and p_VOE=_0.70) and Swin-Unet (p_DSC_=0.09, p_SE_=0.13 and p_VOE_=0.83). For JSC, it shows significant difference from U-Net (*p* = 0.05) and Swin-Unet (*p* = 0.02), but no significant difference from Attention U-Net (*p* = 0.90) and TransUNet (*p* = 0.08). For PPV, it shows significant difference from the three control group models (U-Net (*p* = 0.01), TransUNet (*p* < 0.001), and Swin-UNet (*p* = 0.03)), except Attention-UNet (*p* = 0.08). For HD and RVD, it presents significant difference from TransUNet (both *p* < 0.001), but no significant difference from U-Net (p_HD_=0.23 and p_RVD_=0.16), Attention U-Net (p_HD_=0.63 and p_RVD_=0.86) and Swin-Unet (p_HD_=0.18 and p_RVD_=0.54). Compared with the comparative models, MSAU-Net V2 obtained all the best metrics: DSC of 0.71 ± 0.28, JSC of 0.61 ± 0.27, PPV of 0.85 ± 0.22, SE of 0.68 ± 0.30, HD of 4.04 ± 1.49, RVD of 0.32 ± 0.31 and VOE of 0.45 ± 0.56. As shown in Supplementary materials Part 8, LSD analysis shows that for PPV, our proposed MSAU-Net V2 exhibits significant difference from all the comparative models (p_U−Net_< 0.001, p _Attention U−Net_=0.04, p_TransUNet_ <0.001, p_Swin−Unet_ =0.01). For DSC and JSC, MSAU-Net V2 presents significant difference from U-Net (both *p* < 0.001), TransUNet (p_DSC_ = 0.03 and p_JSC_ < 0.001) and Swin-Unet (both *p* < 0.001), but no difference from Attention U-Net (p_DSC=_0.12 and p_JSC_=0.18). For SE, MSAU-Net V2 shows significant difference from U-Net (*p* < 0.001) and Swin-Unet (*p* < 0.001), but no difference from Attention-U-Net(p_SE=_0.20) and TransUNet (p_SE_=0.81). For RVD, MSAU-Net V2 shows significant difference from U-Net (*p* = 0.02) and TransUNet (*p* < 0.001), but no difference from Attention U-Net (p_RVD_ =0.23) and Swin-Unet (p_RVD_=0.10).

The comprehensive analysis of the external experimental results has exhibited that the proposed MSAU-Net V2 model exhibits significant advantages across multiple metrics, possessing good generalization ability and robustness, proving its better suitableness for segmentation tasks with complex data in real clinical scenarios.

## Discussion

This study introduces a novel deep learning module designed to enhance the U-Net architecture for automated delineation of the gallbladder. Both quantitative and qualitative results indicate that our models significantly improve performance, especially in identifying and delineating fine structures and tissues around the gallbladder. This enhanced capability is primarily attributed to the integration of multi-scale feature extraction and multi-scale spatial attention mechanisms. The multi-scale spatial attention mechanism particularly enhances the model’s focus on relevant features, while effectively suppressing noise and irrelevant details. This approach enables the model to gather information from different scales of feature maps, allowing for a comprehensive capture of both macroscopic and microscopic details which traditional single-scale attention mechanisms might overlook. Consequently, it renders the possibility of a more comprehensive analysis of imaging data, significantly benefiting the segmentation of complex structures such as the gallbladder.

Moreover, the use of multi-scale convolutional kernels for feature map extraction ensures that the model obtains robust global and local information. This approach is crucial for feeding into the spatial attention mechanisms and can ensure that the model focuses on the most relevant features across all scales. This ability to integrate and analyze information from various spatial contexts substantially enhances the model’s precision in delineating the boundaries of the gallbladder, setting a new standard for accuracy in medical imaging segmentation.

Additionally, studies indicate that a DSC greater than 0.7 is generally considered acceptable for medical image segmentation results [27–30]. Our proposed models both achieved the DSC of 0.79, substantially surpassing this industry benchmark. This achievement not only underscores the excellent performance of our models in accurately segmenting the gallbladder but also confirms their reliability and applicability in clinical settings.

Comparative analyses with state-of-the-art models further validate the enhancements brought by the MSSA mechanisms integrated into our MSAU-Net models. Lin et al. [31]. previously introduced the V-Attention U-Net for segmenting OARs in abdominal images, including the gallbladder. According to their findings, the V-Attention U-Net achieved a DSC of 0.76 ± 0.20 and a Hausdorff Distance (HD) of 7.21 ± 13.03, demonstrating considerable segmentation efficacy. In comparison, our models, MSAU-Net V1 and V2 significantly outperformed their proposed model. Both versions achieved a DSC of 0.79, indicating a more precise segmentation capability. Moreover, the HD values for MSAU-Net V1 and V2 were 0.37 ± 0.53 and 0.32 ± 0.42, respectively, substantially lower than those recorded for the V-Attention U-Net, highlighting a marked improvement in boundary accuracy. Additionally, in a related study by Zhang et al. [32], the SequentialSegNet designed for abdominal organ segmentation yielded a DSC of 0.69 ± 0.22 for gallbladder segmentation. Compared to the baseline, the two models, MSAU-Net V1 and V2 achieved an improvement by approximately 14.49%. This indicates a significant enhancement by our proposed models and proves that our models still present good segmentation when confronted with the complex anatomical structure of the gallbladder and its complicated surrounding tissues, further highlighting our models’ robustness and generalization.

There exist some limitations in our study, particularly about application into multiple modalities and multiple medical settings, which influence the model’s robustness and generalization. Diversity in imaging conditions and variety of clinical settings may provoke discrepancies in image characteristics, thus challenging the model’s performance. Differences in resolution, contrast, and modalities such as MRI, and ultrasound may require significant adaptation of the model. Furthermore, the diverse range of equipment, procedural protocols, and patient demographics encountered in clinical environments introduces numerous variables that can potentially impact the generalizability of the model. Further exploration of our proposed model’s validation in multiple modalities and extensive clinical settings will be further conducted in the future study. This will involve collecting more diverse datasets encompassing various imaging modalities and clinical environments, as well as developing cross-modality learning techniques, such as domain adaptation and transfer learning, to mitigate discrepancies between modalities so as to reduce the training time and complexity and increase generalization in different clinical applications. Additionally, integrating advanced data augmentation strategies tailored to specific modalities may further enhance the model’s robustness. Another noteworthy point is that the advancement of general-purpose models promoted universal segmentation frameworks like the Segment Anything Model (SAM) to serve as potential substitutes for manual delineation and demonstrate remarkable generalization capabilities in natural image segmentation tasks. However, the complexity and specificity inherent in medical imaging render considerable challenges for their direct application in specialized medical domain. In recent years, researchers have modified and adapted these models for medical applications, which could potentially emerge as a promising solution to multimodal challenges in the future.

By overcoming these challenges, our proposed models can be expanded to accommodate broader clinical practices, facilitating its adoption in more diverse medical applications and ultimately improving its utility in real-world scenarios.

## Conclusion

The MSAU-Net V1 and V2 both exhibited outstanding performance in gallbladder segmentation, demonstrating strong potential for clinical application, with the optimal number of MSA being three for V1 and two for V2. The MSA block enhances spatial information capture, improving the model’s ability to segment small and complex structures with greater precision. These advantages position MSAU-Net V1 and V2 as valuable tools for broader clinical adoption, withV2 demonstrating better efficacy and potential for multicenter and future clinical promotion.

## Electronic supplementary material

Below is the link to the electronic supplementary material.

Supplementary Material 1

## Data Availability

The datasets generated and/or analysed during the current study are not publicly available due to protection of patient privacy but are available from the corresponding author on reasonable request.
