# Supplementary material for: Multi-spatial-attention U-Net: a novel framework for automated gallbladder segmentation on CT images
Source: BMC Med Imaging. 2025 May 30;25:197. doi: 10.1186/s12880-025-01737-7 (PMC12125801; doi:10.1186/s12880-025-01737-7)
Supplement: Supplementary file 1 — Supplementary Material 1 [file 12880_2025_1737_MOESM1_ESM.docx]

Multi-Spatial-Attention U-Net: A Novel Framework for Automated Gallbladder Segmentation on CT Images

**Supplementary Materials**

**Part 1: CT scan parameters and enhancement methods**

**1.1 CT scan parameters and enhancement methods for data from Qingdao University Affiliated Hospital**

The CT examinations, both contrast-enhanced and non-enhanced, were conducted using a multidetector CT scanner comprising Optima CT670 from GE Healthcare, iCT 256 from Philips Healthcare, and SOMATOM Definition Flash from Siemens Medical Systems. All patients assumed the supine position during the scan, which encompassed either the upper abdomen or the entire abdomen. Patients were instructed to hold their breath while being scanned. For contrast-enhanced examinations, intravenous 1.0 mL/kg contrast medium (iohexol injection, 300 mg/mL, Beilu Pharmaceutical Co. Ltd., Beijing, China) was injected at a flow rate of 3.0–3.5 mL/s using a power injector (Ulrich CT Plus 150, Ulrich Medical), followed by a saline flush (20 mL). Arterial phase scanning was performed with a post-injection delay of 30 seconds, while venous phase scanning had a delay of 70 seconds，and delayed phase scanning had a delay of 2 seconds. The CT scanning parameters included automatic tube current and tube voltage set at 120 kV, field of view ranging between 35-50 cm, detector collimation options of either 64 × 0.6 mm or 128 × 0.625 mm, matrix size set at 512 ×512, slice thickness and interval maintained at 5 mm, and reconstructed section thickness varying between 0.625-2 mm.

**1.2 CT scan parameters and enhancement methods for data from People’s Hospital of Rizhao**

The CT examinations, both contrast-enhanced and non-enhanced, were conducted using a multidetector CT scanner of SOMATOM Definition Flash Dual-Source from Siemens Medical Systems. All patients assumed the supine position during the scan, which encompassed either the upper abdomen or the entire abdomen. Patients were instructed to hold their breath while being scanned. For contrast-enhanced examinations, intravenous 1.0 mL/kg contrast medium (iodixanol injection, 320 mg/mL, GE Healthcare AS) was injected at a flow rate of 3.0–3.5 mL/s using a power injector (Ulrich CT Plus 150, Ulrich Medical), followed by a saline flush (18mL). Arterial phase scanning was performed with a post-injection delay of 30 seconds, while venous phase scanning had a delay of 60 seconds，and delayed phase scanning had a delay of 90 seconds. The CT scanning parameters included automatic tube current and tube voltage set at 120 kV, field of view ranging between 35-50 cm, detector collimation options of 128 × 0.6 mm, matrix size set at 512 ×512,slice thickness and interval maintained at 5 mm,and reconstructed section thickness varying between 0.625-2 mm.

**Part 2: Data Augmentation Techniques**

In this study, various data augmentation techniques were employed to enhance the robustness and generalization of our models. Specifically, the augmentation procedures included rotation, shift, shear, and zoom. For rotation, images were randomly rotated within a range of ±2 degrees to simulate varying orientations. The shift parameter was set at 0.01 for both horizontal and vertical translations. The shear transformation was applied with a parameter of 0.01. Similarly, zooming was conducted with a parameter setting of 0.01. These controlled transformations were systematically applied to augment the training dataset, thereby enabling the models to learn more diverse features from the medical images.

**Part 3:** Architectures of MSAU-Net with sSE

As illustrated in Figure 1，MSAU-Net with sSE are transformed into V1 （with one Multi-Scale Feature Extraction and Fusion (MSFEF) module and V2 （with two MSFEF modules).

V1 employs multi-scale convolutional kernel (3×3, 5×5, 7×7) to extract multi-scale features. It first reduces the channel dimension to C/2 using a 1×1 convolution to decrease computational cost, then restores it to the original dimension C, and finally fuses multi-branch features via skip connections. Finally, it incorporates a channel squeeze and spatial Excitation ( sSE) module to generate spatial attention weights, enhancing features in key regions. V2 further expands the convolutional kernel size to 9×9, enabling a larger global receptive field for improved feature extraction.


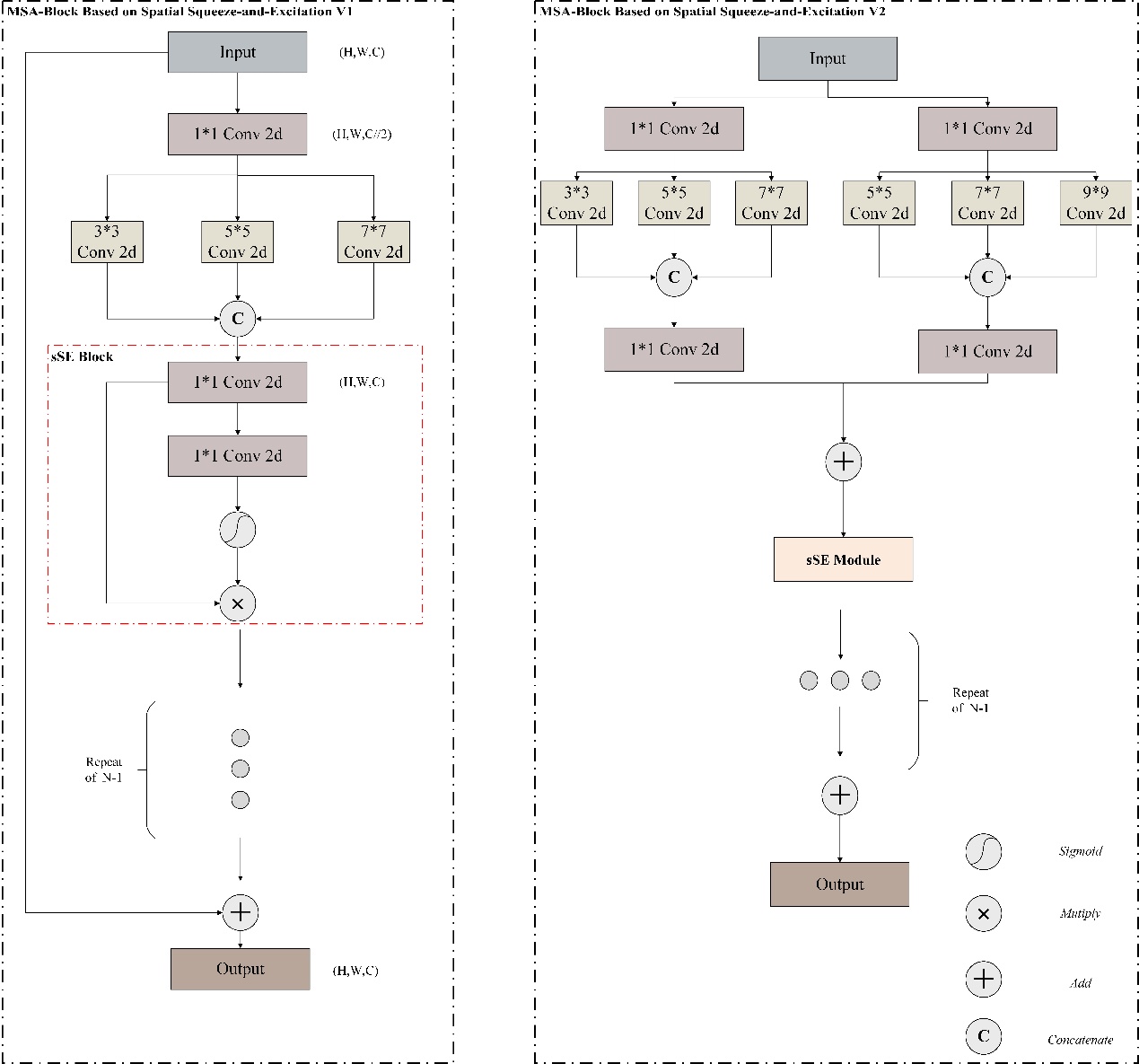


**Figure 1.** A**.** MSA block in MSAU-Net V1 B**.** MSA block in MSAU-Net V2

**Part 4: Model hyperparameter**

In this study, the input CT images for gallbladder segmentation are grayscale images of size 512×512×1. Each image undergoes several convolutional transformations with the exception of the output from the last up-sampling layer. This layer employs a 1×1 2D convolution paired with a Sigmoid activation function to determine whether each pixel in the image represents gallbladder tissue. Throughout the rest of the network, a ReLU activation function is utilized after each convolutional operation to introduce non-linearity and enhance feature learning.

To ensure accurate fusion of features from corresponding down-sampling and up-sampling layers, it is essential to maintain consistent image resolution during the feature fusion process. Thus, zero padding is incorporated during convolutional operations to keep the image size unchanged at each resolution level. This approach obviates the need for cropping in the skip connection process.

The remaining hyperparameters are listed in Table 1. **Batch Size** indicates the number of training samples processed before the model's internal parameters are updated. **Block Number** specifies the count of layers or blocks within the network, such as Multi-Scale Spatial Attention (MSSA) or spatial squeeze-and-excitation (sSE) blocks, affecting the model’s depth and complexity in feature extraction. **Epoch** count represents how many times the entire dataset is passed through the neural network, crucial for the thorough training of the model. **Learning Rate** is vital for determining the step size at each iteration towards minimizing the loss function, while **Decay Steps** and **Decay Rate** manage the frequency and scale at which the learning rate is reduced, allowing for finer adjustments and more stable convergence as training progresses.

**Supplementary Material Table1 Network Training parameters**

|  | ***Model*** | ***Batch Size*** | | ***Block Number*** | | ***Epoch*** | ***Learning Rate*** | ***Decay Steps*** | ***Decay Rate*** | | |
| --- | --- | --- | --- | --- | --- | --- | --- | --- | --- | --- | --- |
|  | U-Net | | 2 | |  | 80 | 2e-4 |  | | |  |
|  | Attention U-Net | | 2 | |  | 80 | 6e-5 | 1200 | | 0.96 | |
|  | TransUNet | | 2 | |  | 80 | 6e-4 | 1200 | | 0.96 | |
|  | Swin-Unet | | 2 | |  | 80 | 1e-5 | 2800 | | 0.96 | |
|  | MSAU-Net V1  (sSE) | | 2 | | 2 | 80 | 1e-4 | 800 | | 0.96 | |
|  | MSAU-Net V1  (sSE) | | 2 | | 3 | 80 | 2e-4 | 800 | | 0.96 | |
|  | MSAU-Net V1  (sSE) | | 2 | | 4 | 80 | 1e-4 | 800 | | 0.96 | |
|  | MSAU-Net V1  (MSSA) | | 2 | | 2 | 80 | 2e-4 | 800 | | 0.96 | |
|  | MSAU-Net V1  (MSSA) | | 2 | | 3 | 80 | 4e-5 | 800 | | 0.96 | |
|  | MSAU-Net V1  (MSSA) | | 2 | | 4 | 80 | 4e-4 | 800 | | 0.96 | |
|  | MSAU-Net V2  (sSE) | | 2 | | 2 | 80 | 3e-4 | 800 | | 0.96 | |
| MSAU-Net V2  (sSE) | | | 2 | | 3 | 80 | 4e-4 | 800 | | 0.96 | |
|  | MSAU-Net V2  (MSSA) | | 2 | | 2 | 80 | 2e-4 | 800 | | 0.96 | |
|  | MSAU-Net V2  (MSSA) | | 2 | | 3 | 80 | 5e-5 | 1200 | | 0.96 | |

**Part 5: Model Environment**

The model was built using TensorFlow software version 2.4.0 (Google Brain Team,2015; Mountain View, CA, USA) and Keras software version 2.4.3 (Chollet, 2015) ,with Python 3 (Van Rossum and Drake, 2009) as the programming language, Windows 10 64-bit as operating system (Microsoft Corp.,Redmond, WA, USA), Intel Core i9-10900 KF @ 3.70 GHz (Intel Corp., Santa Clara, CA, USA)as the CPU, NVIDIA GTX3090 24 G (NVIDIA Corp., Santa Clara, CA, USA) as the graphics card, and 128GB memory.

**Part 6： Evaluation Metrics for Segmentation Accuracy and Generalization**

In this study, we utilized several common evaluation metrics in medical image segmentation to assess the segmentation accuracy and generalization capability of the models. The evaluation metrics include Dice Similarity Coefficient (DSC), Jaccard Similarity Coefficient (JSC), Positive Predictive Value (PPV), Sensitivity (SE), Hausdorff Distance (HD), Relative Volume Difference (RVD), and Volume Overlap Error (VOE). The mathematical definitions of these metrics are provided below.

**6.1. Dice Similarity Coefficient (DSC)**
DSC quantifies the overlap between the predicted segmentation and the ground truth by calculating the ratio of twice the intersection over the sum of their pixel counts.

,

The DSC value ranges from 0 to 1, with values closer to 1 indicating a higher degree of overlap and, thus a better prediction performance.

**6.2. Jaccard Similarity Coefficient (JSC)**

JSC measures the ratio of the intersection to the union of the predicted segmentation and the ground truth.

,

Similar to DSC, the JSC ranges from 0 to 1, with values closer to 1 representing better segmentation results.

**6.3. Positive Predictive Value (PPV) and Sensitivity (SE)**

PPV, or precision, calculates the fraction of true positive pixels out of all pixels predicted as part of the target structure. SE, also known as recall, represents the proportion of actual positive pixels correctly identified by the model.

,

,

**TP**: Correctly predicted positive pixels. **FP**: Background pixels incorrectly predicted as positive.

**FN**: Actual positive pixels incorrectly predicted as background.

**6.4.** **Hausdorff Distance (HD)**

HD is the maximum distance between the boundary points of the predicted segmentation and the closest boundary points in the ground truth. It provides a measure of spatial boundary accuracy.

,

in which ，, and the smaller HD indicates better prediction.

**6.5. Relative Volume Difference (RVD) and Volume Overlap Error (VOE)**

RVD measures the relative discrepancy in volume between the predicted segmentation and the ground truth. VOE calculates the proportion of non-overlapping volume between the predicted and ground truth regions, assessing the segmentation error in terms of volumetric overlap.

,

,

RVD Values close to 0 indicate a smaller volume difference, representing a more accurate prediction. Lower VOE values indicate better overlap between the prediction and the ground truth.

**Part 7：**Tables (1-4) for the quantitative evaluation for MSAU-Net V1 and V2 models with different MSA block counts based on the sSE or MSSA mechanisms.

The results reveal that the optimal performance, in terms of DSC, JSC, PP, SE, HD, RVD, and VOE, is generally achieved when the module count is set to either 2 or 3. For MSAU-Net V1 with sSE (Table 2), N=2 provides the best performance, while increasing to N=3 or N=4 leads to diminishing returns, particularly in DSC and JSC. A similar trend is observed in MSAU-Net V1 with MSSA (Table 3), where N=2 and N=3 offer comparable performance, but a noticeable decline is seen at N=4. For MSAU-Net V2, Table 4 shows that the model with sSE achieves improved results with N=3, and Table 5 demonstrates that the MSSA-based MSAU-Net V2 performs best with N=2. These results suggest that while increasing the number of modules may improve the model's ability to extract features and capture spatial information, overcomplicating the model with too many modules can reduce performance due to increased complexity and potential overfitting. Therefore, finding the right balance in module count is essential to maintain high accuracy without sacrificing computational efficiency or risking overfitting.

**Supplementary Material Table 1: Quantitative evaluation for different MSA block counts of MSAU-Net model V1 with sSE**

| Metric | N=2 | N=3 | N=4 |
| --- | --- | --- | --- |
| DSC | **0.76±0.27** | 0.75±0.27 | 0.67±0.28 |
| JSC | **0.67±0.28** | 0.66±0.28 | 0.56±0.29 |
| PPV | **0.85±0.20** | 0.79±0.25 | 0.71±0.29 |
| SE | 0.76±0.29 | **0.78±0.28** | 0.70±0.29 |
| HD | **3.33±1.29** | 3.47±1.30 | 3.90±1.44 |
| RVD | **0.37±0.49** | 0.39±0.52 | 0.53±0.73 |
| VOE | 0.45±0.59 | **0.37±0.45** | 0.46±0.46 |

**Supplementary Material Table 2: Quantitative evaluation for different MSA block counts of MSAU-Net model V1 with MSSA**

| Metric | N=2 | N=3 | N=4 |
| --- | --- | --- | --- |
| DSC | 0.78±0.27 | **0.79±0.25** | 0.76±0.26 |
| JSC | 0.70±0.27 | **0.70±0.25** | 0.67±0.27 |
| PPV | 0.85±0.20 | **0.85±0.20** | 0.80±0.26 |
| SE | 0.80±0.28 | 0.80±0.26 | **0.79±0.26** |
| HD | **3.22±1.29** | 3.23±1.23 | 3.38±1.28 |
| RVD | 0.37±0.56 | **0.33±0.45** | 0.39±0.61 |
| VOE | 0.39±0.55 | 0.37±0.53 | **0.34±0.44** |

**Supplementary Material Table 3: Quantitative evaluation for different MSA block counts of MSAU-Net model V2 with sSE**

| Metric | N=2 | N=3 |
| --- | --- | --- |
| DSC | 0.64±0.35 | **0.73±0.28** |
| JSC | 0.56±0.33 | **0.63±0.28** |
| PPV | 0.78±0.30 | **0.80±0.26** |
| SE | 0.63±0.36 | **0.72±0.28** |
| HD | 3.70±1.32 | **3.44±1.23** |
| RVD | 0.42±0.46 | **0.40±0.54** |
| VOE | 0.54±0.63 | **0.40±0.48** |

**Supplementary Material Table 4: Quantitative evaluation for different MSA block counts of MSAU-Net model V2 with MSSA**

| Metric | N=2 | N=3 |
| --- | --- | --- |
| DSC | **0.79±0.24** | 0.75±0.27 |
| JSC | **0.70±0.25** | 0.66±0.28 |
| PPV | **0.83±0.23** | 0.81±0.26 |
| SE | **0.80±0.25** | 0.76±0.28 |
| HD | **3.20±1.23** | 3.35±1.26 |
| RVD | **0.34±0.53** | 0.37±0.54 |
| VOE | **0.32±0.42** | 0.39±0.51 |

**Part 8：**Tables 5 and 6 exhibit the results of internal and external validations of MSAU-NetV1 and V2 in comparison with the control group models. The detailed description of the results can be found in the manuscript.

**Supplementary Material Table 5: LSD pair comparison between** **MSAU-Net V1 and comparison models & MSAU-Net V2 and comparison models**

| Metrics | Our model |  | Control group | Sig. |
| --- | --- | --- | --- | --- |
| DSC | **MSAU-Net V1** |  | U-Net | <0.001 |
|  |  |  | Attention U-Net | .04 |
|  |  |  | MSAU-Net V2 | .91 |
|  |  |  | TransUNet | <0.001 |
|  |  |  | Swin-Unet | <0.001 |
|  | **MSAU-Net V2** |  | U-Net | <0.001 |
|  |  |  | Attention U-Net | .06 |
|  |  |  | MSAU-NET V1 | .91 |
|  |  |  | TransUnet | <0.001 |
|  |  |  | Swin-Unet | <0.001 |
| JSC | **MSAU-NET V1** |  | U-Net | <0.001 |
|  |  |  | Attention U-Net | .09 |
|  |  |  | MSAU-Net V2 | .82 |
|  |  |  | TransUnet | <0.001 |
|  |  |  | Swin-Unet | <0.001 |
|  | **MSAU-Net V2** |  | U-Net | <0.001 |
|  |  |  | Attention U-Net | .14 |
|  |  |  | MSAU-NET V1 | .82 |
|  |  |  | TransUnet | <0.001 |
|  |  |  | Swin-Unet | <0.001 |
| PPV | MSAU-NET V1 |  | U-Net | .03 |
|  |  |  | Attention U-Net | .14 |
|  |  |  | MSAU-Net V2 | .98 |
|  |  |  | TransUnet | <0.001 |
|  |  |  | Swin-Unet | <0.001 |
|  | MSAU-Net V2 |  | U-Net | .03 |
|  |  |  | Attention U-Net | .15 |
|  |  |  | MSAU-NET V1 | .98 |
|  |  |  | TransUnet | <0.001 |
|  |  |  | Swin-Unet | <0.001 |
| SE | MSAU-NET V1 |  | U-Net | <0.001 |
|  |  |  | Attention U-Net | .03 |
|  |  |  | MSAU-Net V2 | .75 |
|  |  |  | TransUnet | .58 |
|  |  |  | Swin-Unet | <0.001 |
|  | MSAU-Net V2 |  | U-Net | <0.001 |
|  |  |  | Attention U-Net | .06 |
|  |  |  | MSAU-NET V1 | .75 |
|  |  |  | TransUnet | .81 |
|  |  |  | Swin-Unet | <0.001 |
| HD | MSAU-NET V1 |  | U-Net | .16 |
|  |  |  | Attention U-Net | .55 |
|  |  |  | MSAU-Net V2 | .66 |
|  |  |  | TransUnet | <0.001 |
|  |  |  | Swin-Unet | <0.001 |
|  | MSAU-Net V2 |  | U-Net | .06 |
|  |  |  | Attention U-Net | .30 |
|  |  |  | MSAU-NET V1 | .66 |
|  |  |  | TransUnet | <0.001 |
|  |  |  | Swin-Unet | <0.001 |
| RVD | MSAU-NET V1 |  | U-Net | .05 |
|  |  |  | Attention U-Net | .32 |
|  |  |  | MSAU-Net V2 | .74 |
|  |  |  | TransUnet | <0.001 |
|  |  |  | Swin-Unet | <0.001 |
|  | MSAU-Net V2 |  | U-Net | .10 |
|  |  |  | Attention U-Net | .51 |
|  |  |  | MSAU-NET V1 | .74 |
|  |  |  | TransUnet | <0.001 |
|  |  |  | Swin-Unet | <0.001 |
| VOE | MSAU-NET V1 |  | U-Net | <0.001 |
|  |  |  | Attention U-Net | .01 |
|  |  |  | MSAU-Net V2 | .56 |
|  |  |  | TransUnet | <0.001 |
|  |  |  | Swin-Unet | <0.001 |
|  | MSAU-Net V2 |  | U-Net | <0.001 |
|  |  |  | Attention U-Net | <0.001 |
|  |  |  | MSAU-NET V1 | .56 |
|  |  |  | TransUnet | <0.001 |
|  |  |  | Swin-Unet | <0.001 |

**Supplementary Material Table 6: LSD pair comparison between MSAU-Net V1 and comparison models & MSAU-Net V2 and comparison models for external validation**

| Metrics | Our model | Control group | Sig. |
| --- | --- | --- | --- |
| DSC | **MSAU-Net V1** | U-Net | .04 |
|  |  | Attention U-Net | .78 |
|  |  | MSAU-Net V2 | .20 |
|  |  | TransUNet | .36 |
|  |  | Swin-Unet | .09 |
|  | **MSAU-Net V2** | U-Net | <0.001 |
|  |  | Attention U-Net | .12 |
|  |  | MSAU-NET V1 | .20 |
|  |  | TransUNet | .03 |
|  |  | Swin-Unet | <0.001 |
| JSC | **MSAU-NET V1** | U-Net | .05 |
|  |  | Attention U-Net | .90 |
|  |  | MSAU-Net V2 | .22 |
|  |  | TransUNet | .08 |
|  |  | Swin-Unet | .02 |
|  | **MSAU-Net V2** | U-Net | <0.001 |
|  |  | Attention U-Net | .18 |
|  |  | MSAU-NET V1 | .22 |
|  |  | TransUNet | <0.001 |
|  |  | Swin-Unet | <0.001 |
| PPV | MSAU-NET V1 | U-Net | .01 |
|  |  | Attention U-Net | .08 |
|  |  | MSAU-Net V2 | .76 |
|  |  | TransUNet | <0.001 |
|  |  | Swin-Unet | .03 |
|  | MSAU-Net V2 | U-Net | <0.001 |
|  |  | Attention U-Net | .04 |
|  |  | MSAU-NET V1 | .76 |
|  |  | TransUNet | <0.001 |
|  |  | Swin-Unet | .01 |
| SE | MSAU-NET V1 | U-Net | .02 |
|  |  | Attention U-Net | .92 |
|  |  | MSAU-Net V2 | .17 |
|  |  | TransUNet | .25 |
|  |  | Swin-Unet | .13 |
|  | MSAU-Net V2 | U-Net | <0.001 |
|  |  | Attention U-Net | .20 |
|  |  | MSAU-NET V1 | .17 |
|  |  | TransUNet | .81 |
|  |  | Swin-Unet | <0.001 |
| HD | MSAU-NET V1 | U-Net | .23 |
|  |  | Attention U-Net | .63 |
|  |  | MSAU-Net V2 | .72 |
|  |  | TransUNet | <0.001 |
|  |  | Swin-Unet | .18 |
|  | MSAU-Net V2 | U-Net | .12 |
|  |  | Attention U-Net | .40 |
|  |  | MSAU-NET V1 | .72 |
|  |  | TransUNet | <0.001 |
|  |  | Swin-Unet | .09 |
| RVD | MSAU-NET V1 | U-Net | .16 |
|  |  | Attention U-Net | .86 |
|  |  | MSAU-Net V2 | .31 |
|  |  | TransUNet | <0.001 |
|  |  | Swin-Unet | .54 |
|  | MSAU-Net V2 | U-Net | .02 |
|  |  | Attention U-Net | .23 |
|  |  | MSAU-NET V1 | .31 |
|  |  | TransUNet | <0.001 |
|  |  | Swin-Unet | .10 |
| VOE | MSAU-NET V1 | U-Net | .03 |
|  |  | Attention U-Net | .89 |
|  |  | MSAU-Net V2 | .11 |
|  |  | TransUNet | .70 |
|  |  | Swin-Unet | .83 |
|  | MSAU-Net V2 | U-Net | <0.001 |
|  |  | Attention U-Net | .15 |
|  |  | MSAU-NET V1 | .11 |
|  |  | TransUNet | .23 |
|  |  | Swin-Unet | .17 |
